# SentiMedQAer: A Transfer Learning-Based Sentiment-Aware Model for Biomedical Question Answering

**DOI:** 10.3389/fnbot.2022.773329

**Published:** 2022-03-10

**Authors:** Xian Zhu, Yuanyuan Chen, Yueming Gu, Zhifeng Xiao

**Affiliations:** ^1^School of Information Management, Nanjing University, Nanjing, China; ^2^School of Health Economics and Management, Nanjing University of Chinese Medicine, Nanjing, China; ^3^Centre for Data Science, Institute of Collaborative Innovation, University of Macau, Macau, China; ^4^School of Computing and Information Systems, Faculty of Engineering and Information Technology, University of Melbourne, Parkville, VIC, Australia; ^5^School of Engineering, Penn State Erie, The Behrend College, Erie, PA, United States

**Keywords:** biomedical question answering, T5, RoBERTa, sentiment analysis, transfer learning, XGBoost

## Abstract

Recent advances have witnessed a trending application of transfer learning in a broad spectrum of natural language processing (NLP) tasks, including question answering (QA). Transfer learning allows a model to inherit domain knowledge obtained from an existing model that has been sufficiently pre-trained. In the biomedical field, most QA datasets are limited by insufficient training examples and the presence of factoid questions. This study proposes a transfer learning-based sentiment-aware model, named SentiMedQAer, for biomedical QA. The proposed method consists of a learning pipeline that utilizes BioBERT to encode text tokens with contextual and domain-specific embeddings, fine-tunes Text-to-Text Transfer Transformer (T5), and RoBERTa models to integrate sentiment information into the model, and trains an XGBoost classifier to output a confidence score to determine the final answer to the question. We validate SentiMedQAer on PubMedQA, a biomedical QA dataset with reasoning-required yes/no questions. Results show that our method outperforms the SOTA by 15.83% and a single human annotator by 5.91%.

## 1. Introduction

Retrieving high-quality short answers to a given natural language question from the growing biomedical literature is key to creating high-quality systematic evaluations that support evidence-based medical practice (Stylianou et al., 2020) and improve the quality of patient care (Kumbhakarnaa et al., 2020). However, the explosion in the volume of scientific literature in biomedicine makes it difficult for even experts in their field of interest to assimilate all relevant information. As a result, there is an increasing number of studies that require more sophisticated techniques and automated biomedical text mining methods in order to provide relevant answers to information seekers. Current venues that aggregate scientific advances in biomedicine are mainly search engines based on information retrieval (IR) (Singhal et al., 2001) techniques, such as PubMed and Google Scholar. However, in the current setup, the size of the answers represented by the retrieved set of documents (which may be relevant) is still too large to easily identify precise information. Users often have to manually examine and filter the returned documents to find the exact information they are looking for. A study by Hersh et al. (2002) showed that medical and nurse practitioner students took an average of at least 30 min to answer clinical questions using MEDLINE. Unlike IR, question and answer (QA) (Diefenbach et al., 2018) systems can generate and provide accurate answers directly to users' questions in less than a few seconds by automatically analyzing thousands of articles, thus freeing users from the time-consuming literature screening.

Building an efficient and accurate biomedical QA system is challenging. Athenikos and Han (2010) have highlighted three characteristics of biomedical QA: (1) large text corpora, (2) highly complex domain-specific terminology, and (3) domain-specific formats and question types. Meanwhile, the current biomedical QA task suffers from the following difficulties. First, the low-resource setting of biomedical QA limits the performance of deep models. Second, the knowledge gap between biomedical experts and machine learning experts brings difficulty in integrating biomedical domain knowledge into predictive models. Lastly, biomedicine is evolving rapidly, models developed for old tasks may not suit the needs of emerging tasks (Jin et al., 2021).

The major problem of existing biomedical QA datasets is the limited size caused by the high cost of manual annotation (Jin et al., 2021). For example, a widely used dataset, BioASQ (Tsatsaronis et al., 2015), has less than 3,000 training examples. Annotation automation techniques have also applied to build large scale biomedical QA datasets, and examples include BioRead (Pappas et al., 2018), BMKC (Kim et al., 2018), and emrQA (Pampari et al., 2018). The first two belong to cloze-style QA, which requires a model to predict the masked bio-entities. EmrQA is created by a novel method by re-purposing existing annotations for other NLP tasks; the resulting corpus consists of 400,000 QA pairs with evidence. However, current efforts of auto-generation of biomedical QA samples mainly focus on factoid questions, with answers that can be directly extracted from the given context, which does not work well for QA systems in need of reasoning over the context. To address this problem, a recent effort by Jin et al. present PubMedQA (Jin et al., 2019), a yes/no QA dataset created from PubMed abstracts. Unlike prior datasets, questions in PubMedQA are titles or derived from titles, and the contexts are abstract bodies. To predict an answer, a model needs to reason over the context, establishing a semantic connection between a question and a context.

- We propose a sentiment-aware model named SentiMedQAer that learns to answer yes/no questions given a biomedical context. SentiMedQAer consists of a custom learning pipeline that (1) uses BioBERT to encode tokens with contextual and domain-specific embeddings, (2) fine-tunes Text-to-Text Transfer Transformer (T5) and RoBERTa to integrate sentiment information into the learning task, and (3) lastly adopts an XGBoost model to learn a confidence score that can determine the likely answer to the question.
- The proposed model is validated on the PubMedQA dataset and outperforms the SOTA by 15.83% in accuracy and outperforms a single human annotator by 5.91%, demonstrating the efficacy of sentiment tendency in the task of yes/no biomedical QA.

The rest of this article is structured as follows. A collection of relevant studies are reviewed in Section 2. A description of the dataset and the technical details of the proposed method are provided in Section 3. We report the experimental settings, implementation, and the results in Section 4. Lastly, Section 5 concludes the work with potential directions for future research.

## 2. Related Work

### 2.1. Biomedical QA Datasets

A wide spectrum of open-domain QA datasets (Yang et al., 2015, 2018; Khot et al., 2020) have been developed and have attracted considerable research attention in recent years. However, the quality of biomedical QA datasets is limited mainly by the size of annotated data. BioASQ (Tsatsaronis et al., 2015), a representative biomedical QA dataset, only has less than 3,000 training examples, and most are simple factual QA instances. BioRead (Pappas et al., 2018) and BMKC (Kim et al., 2018) are cloze-style QA (Lewis et al., 2019) datasets that have been created by masking bio-entities in the text and using the rest of the parts as a context to predict the masked entities, similar to masked language modeling used in BERT for self-supervised pre-training (Mao, 2020). Arnold et al. propose Contextual Discourse Vectors (Arnold et al., 2020) for health care document representation, and sentence-level search is performed to find entities and aspects used for resolving queries with short latency. Automated generation of biomedical QA datasets, such as emrQA (Pampari et al., 2018), have also been studied. These automated methods can quickly generate a large dataset, but the generated QA pairs mostly contain factoid questions with answers extracted from the given contexts. This reasoning-free setting is not practical in many real-world QA systems (Thorndike, 1973). To this end, Jin et al. (2019) have developed PubMedQA, a Yes/No type of biomedical QA dataset that contains sufficient annotated QA instances (including 1,000 human-annotated and 2,113,000 artificially generated) extracted from PubMed abstracts; moreover, to answer a question, a model needs to reason over the given context, namely the abstract body, which is a more difficult but practical task. The novelty of PubMedQA is the main reason for us to choose it as the learning task in this study.

### 2.2. Yes/No QA

Yes/No questions have been widely adopted in existing QA datasets, such as HotpotQA (Yang et al., 2018), ShaRC (Saeidi et al., 2018), BioASQ, Natural Questions (Kwiatkowski et al., 2019), PubMedQA, SciFact (Wadden et al., 2020), and BoolQ (Clark et al., 2019). Although the answer is short and binary, it could be challenging to reason over the given context to give an accurate answer. BoolQ is an example that consists of yes/no questions collected in an unconstrained setting using open-domain corpora. Being a domain-specific version of BoolQ, PubMedQA only considers biomedical short texts, namely PubMed abstracts, most of which are semi-structured with parts of aim, method, result, and conclusion.

The yes/no QA task can be intuitively formatted as a binary classification problem, where the input should include the question and the context, and the answer is either yes or no. Feature-based approaches (Somasundaran et al., 2007; Oh et al., 2012) have been investigated over a decade ago, and recently proposed solutions mostly employ deep neural structures (Ye et al., 2019), along with transfer learning (Jin et al., 2019) to optimize the usage of domain knowledge. Pre-trained models, such as ELMo (Peters et al., 2018), ELECTRA (Clark et al., 2020), BERT (Devlin et al., 2018), and its variants (Jin et al., 2019; Liu et al., 2019), have shown superior performance and refreshed the SOTA records. This study extensively leverages transfer learning, including the use of BioBERT (Liu et al., 2019) to build token embeddings and fine-tuning of two T5 models and a RoBERTa model as the essential components of the proposed learning pipeline. Our method posts a 15.83% gain in accuracy compared to the SOTA on the PubMedQA dataset.

### 2.3. Sentiment-Enhanced QA

Sentiment analysis (Feldman, 2013; Zhang et al., 2018) has been a core downstream task in NLP, aiming to detect the emotional tendency in a given text. A few studies have explored the interconnection between sentiment analysis and QA, driven by an intuition that “if something undesirable happens, the reason is also often something undesirable, and if something desirable happens, the reason is also often something desirable,” quoted from Oh et al. (2012). The existence of sentiment correlation between the question, the context, and the answer has been verified in prior efforts (Somasundaran et al., 2007; Ku et al., 2008; Oh et al., 2012; Elalfy et al., 2015; Eskandari et al., 2015; Pang and Ngo, 2015). Somasundaran et al. (2007) propose to use attitude as a part of the feature set to improve QA performance. Similar ideas have been explored in why-QA (Oh et al., 2012), community QA (Elalfy et al., 2015; Eskandari et al., 2015), opinion QA (Ku et al., 2008; Pang and Ngo, 2015), how-QA (Ye et al., 2019), and yes/no QA (Sarrouti and El Alaoui, 2017). Inversely, QA-style sentiment classification has also been investigated (Shen et al., 2018). The most related study to our work is by Sarrouti and El Alaoui (2017), who develop a yes/no answer generator using sentiment word scores. Specifically, their proposed method labels each token of the context with a sentiment score using SentiWordNet (Baccianella et al., 2010), and the global sentiment score of the given context can be calculated and used to predict the answer. The major differences between our work and (Sarrouti and El Alaoui, 2017) are: (1) our transfer learning-based pipeline can effectively integrate biomedical knowledge into the QA system, (2) we utilize the sentiment information of the question, context, long answer, and short answer to determine a prediction jointly, and (3) our work is validated on PubMedQA with more challenging QA instances that require reasoning.

## 3. Materials and Methods

### 3.1. Dataset

The PubMedQA (Jin et al., 2019) dataset is developed by Jin et al. at the University of Pittsburgh in 2019, and it is regarded as the first QA dataset for reasoning about biomedical research texts, in particular their quantitative content, necessary to answer a biomedical question. All sample instances of PubMedQA are collected from PubMed abstracts. The dataset consists of 1k expert-labeled, 61.2k unlabeled, and 211.3k artificially generated QA instances. Each QA instance of PubMedQA includes four components: (1) a question that is either the title of a article or derived from an existing article title, (2) a context, which is the corresponding abstract with the conclusion statement removed, (3) a long answer, which is the conclusion part of the abstract that may or may not answer the question, and lastly (4) a short answer in the form of “yes/no/maybe” that serves as a summary of the conclusion.

The dataset consists of three subsets, denoted as PQA-L (labeled), PQA-U (unlabeled) and PQA-A (artificially generated). PQA-L and PQA-U are created based on PubMed articles that have a question mark in the title and a structured abstract with a conclusion. These articles are placed in a collection called pre-PQA-U. Each article instance in pre-PQA-U consists of three parts: (1) a question, i.e., the original title, (2) a context, i.e., the abstract without the conclusion, and (3) a long answer, i.e., the conclusion in the abstract. Two annotators with biomedical background are employed to label 1,000 instances of pre-PQA-U with yes/no/maybe annotations, producing PQA-L. The annotation process is briefly described as follows: an instance is randomly sampled from pre-PQA-U, an if the instance is answerable with yes/no/maybe, it is sent to the annotators; the first annotator labels the instance based on the question, context, and the long answer, while the second annotator only uses the question and context to label the instance; if both annotators have the same label, the instance, with the label, is added to PQA-L, otherwise, the two annotators attempt to resolve the dispute; if an agreement is reached, the labeled instance is accepted, or the instance is removed, and the annotators move to the next iteration. It is noted that reasoning is highly required for the second annotator since the long answer is not provided. The first annotator, on the other hand, works in a reasoning-free setting, with the long answer available. The remaining unlabeled instances of pre-PQA-U that are yes/no/maybe answerable are added to the PQA-U collection. Lastly, PQA-A is built using a simple heuristic to convert the original title to a question form and generate noisy yes/no labels for the instances. Compared to BioASQ, PubMedQA is larger and is a yes/no/maybe type of non-factual dataset. Table 1 shows the profile information of the three dataset collections, and Table 2 shows an annotated training example. To conduct a fair comparison, this study follows the data split setting utilized in the original article of PubMedQA (Jin et al., 2019), which divides the 1k human-labeled instances in PQA-L into two equal halves, obtaining a validation and a test set, with 500 instances for each set. This way, we conduct hyperparameter tuning using the validation set and evaluate the model on the test set.

**Table 1:** Profile of PubMedQA.

	**PQA-L**	**PQA-U**	**PQA-A**
# QA pairs	1,000	612,000	2,113,000
Question	O.Q. title	O.Q. title	O. title converted to Q.
Labels	Yes/no/maybe	Unlabeled	Generated yes/no
Yes%	55.20%	-	92.80%
No%	33.80%	-	7.20%
Maybe%	11.00%	-	0

**Table 2:** A sample with annotation in the PubMedQA dataset.

**Article ID**	**23831910**
Question	Double balloon enteroscopy: is it efficacious and safe in a community setting?
Context	**Aim**: Double balloon enteroscopy (DBE) has been extensively used in tertiary referral centers… **Methods**: From March 2007 to January 2011, 88 DBE procedures were performed on 66 patients. Indications included evaluation anemia/gastrointestinal bleed… **Results**: The mean age was 62 years. Thirty-two patients were female, 15 were African-American; 44 antegrade and 44 retrograde DBEs were performed…
Long answer	**Conclusion**: DBE appears to be equally safe and effective when performed in the community setting as compared to a tertiary referral center with a comparable yield, efficacy, and complication rate.
Answer	Yes

### 3.2. Learning Task

Let $M={\left\{m_{i}\right\}}_{i=1}^{N}$ denote a collection of *N* labeled abstracts indexed by *i*. Each abstract can be divided into four parts that make a four-tuple, namely (*q*_*i*_, *c*_*i*_, *l*_*i*_, *a*_*i*_), which represent the question title, the context (i.e., the main abstract body without the conclusion), the conclusion, and the short answer (i.e., yes/no/maybe) to the question. Our learning task here is to train a model with samples (*x*_*i*_, *y*_*i*_), where *x*_*i*_ = (*q*_*i*_, *c*_*i*_, *l*_*i*_), and *y*_*i*_ = *a*_*i*_. After training, when fed with an *x*_*i*_, the model can generate an answer ŷ_*i*_ that approximates the ground truth *y*_*i*_. In addition to this main learning task, there are several auxiliary tasks, defined in the following subsections, that jointly accomplish the main task.

### 3.3. An Overview of the Learning Framework

**Figure 1:**
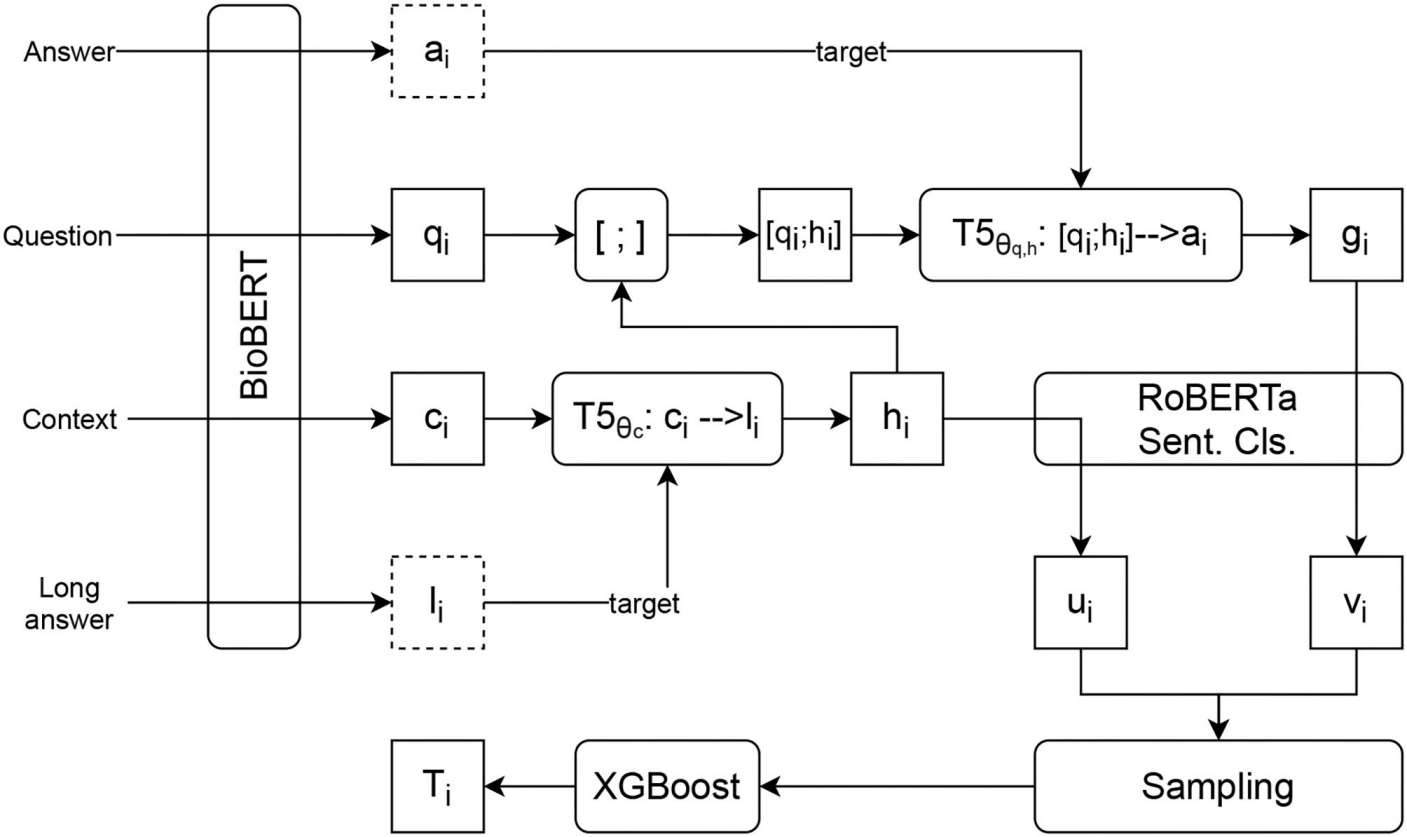
Learning framework of SentiMedQAer: Two T5 models are fine-tuned to produce short forms of the context and the question, which are fed into a RoBERTa model that outputs their sentiment representations. A sampling module is employed to pair these sentiment values utilized to train an XGB classifier, which outputs a confidence score that determines the final prediction result.

Figure 1 presents an overview of the SentiMedQAer learning framework. We fine-tune two T5 networks: one transforms a context *c*_*i*_ to a short form *h*_*i*_, using *l*_*i*_ as the training target; the other transforms a concatenation of *q*_*i*_ and *h*_*i*_ to a short form *g*_*i*_, using *a*_*i*_ as the training target. Taking *h*_*i*_ and *g*_*i*_, a RoBERTa-based sentiment classifier is trained, using *a*_*i*_'s sentiment, *s*_*i*_, as a label. Also, *h*_*i*_ and *g*_*i*_ are fed into the RoBERTa-based sentiment classifier to produce *u*_*i*_ and *v*_*i*_, respectively. Finally, a sampling module is employed to pair *u*_*i*_ and *v*_*i*_, creating sentiment samples to train a XGBoost classifier, which outputs *T*_*i*_, a sentiment confidence score in the range of 0 and 1. *T*_*i*_ is employed to determine the final answer of the question.

### 3.4. BioBERT for Word Embedding

BioBERT (Lee et al., 2020) is a BERT variant pre-trained on PubMed articles for adapting the biomedical domain. There are two typical ways to apply BioBERT to downstream tasks: first, BioBERT can be fine-tuned on a specific dataset to suit the target learning task (Jin et al., 2019); second, BioBERT can be treated as a neural encoder that transforms word tokens of input texts to word embeddings (Naseem et al., 2020). Since BioBERT has been pre-trained on a large biomedical corpus with over a million PubMed articles, it presents superior performance in a variety of biomedical NLP tasks, compared to BERT and other pre-training models (Lee et al., 2020). In this study, we choose the second strategy since BioBERT can generate high-quality contextual embeddings with rich domain knowledge. Each word token is converted to a 768-dimensional embedding. These embeddings serve as input for the next stage in the pipeline.

### 3.5. Fine-Tuning T5

T5 models a wide range of NLP tasks (e.g., machine translation, question answering, text summarization, etc.) as a text-to-text problem. T5 adopts the vanilla transformer with an encoder-decoder structure, and pre-trained on a colossal, cleaned version of Common Crawl's web crawl corpus (c4), which consists of 750GB English texts. The self-supervised pre-training offers three options: predicting the next word (i.e., language modeling), BERT-like objective (predicting the original token that is masked/replaced), and deshuffling (predicting the original text that is rearranged). A recent study has shown the potential of T5 in QA tasks (Zhou and Zhang, 2021) due to its ability to extract key information from the context, where questions and answers are derived from.

The fine-tuning of T5 for our case involves two tasks to transform the long context and question to the short forms that preserve the essential information of a sample. The first task, defined in Equations 1 and 2, fine-tunes T5 with the samples in $D^{c}=\left\{\left(c_{i},l_{i}\right)\right\}$, where *c*_*i*_ is the input, and *l*_*i*_ is the target. The tuned T5, with optimized parameters θ_*c*_, can transform a context *c*_*i*_ to its short form *h*_*i*_, similar to a summary of the context. The second task, given in Equations 3 and 4, fine-tunes T5 with the samples in $D^{qh}=\left\{\left(\left[q_{i};h_{i}\right],a_{i}\right)\right\}$, where the input is [*q*_*i*_; *h*_*i*_], a concatenation of *q*_*i*_ and *h*_*i*_, and the training target is the short answer *a*_*i*_. Another tuned T5, with parameters θ_*qh*_, is obtained to transform [*q*_*i*_; *h*_*i*_] to *g*_*i*_.


(1) $$\begin{array}{l} \theta_{c}\leftarrow \underset{\theta }{\mathrm{argmin}}L\left({\text{T5}}_{\theta }\left(c_{i}\right),l_{i}\right) \end{array}$$

(2) $$\begin{array}{l} h_{i}={\text{T5}}_{\theta_{c}}\left(c_{i}\right) \end{array}$$

(3) $$\begin{array}{l} \theta_{qh}\leftarrow \underset{\theta }{\mathrm{argmin}}L\left({\text{T5}}_{\theta }\left(\left[q_{i};h_{i}\right]\right),a_{i}\right) \end{array}$$

(4) $$\begin{array}{l} g_{i}={\text{T5}}_{\theta_{qh}}\left(\left[q_{i};h_{i}\right]\right) \end{array}$$

### 3.6. Fine-Tuning RoBERTa

RoBERTa (Liu et al., 2019) optimizes the training of BERT with the following strategies: (1) using the same masked language modeling task but removing the next sentence prediction task for self-supervised training; (2) using larger mini batches and learning rates; (3) pre-training on larger corpora with longer time. RoBERTa achieves SOTA on the widely used NLP benchmark, General Language Understanding Evaluation (GLUE).

In this study, we fine-tune RoBERTa to obtain a sentiment estimator, which outputs a sentiment value with a given input message. The task of fine-tuning RoBERTa is formally described in Equations 5 and 6, where *s*_*i*_ is the binary sentiment indicator determined by the short answer *a*_*i*_; if *a*_*i*_ is a “No”, the associated sentiment is negative, otherwise it is positive, as defined in Equation 5. Both *h*_*i*_ and *g*_*i*_ are paired with *s*_*i*_ to form a dataset $D^{gh}=\left\{\left(g_{i},s_{i}\right)\right\}⋃\left\{\left(h_{i},s_{i}\right)\right\}$, used to fine-tune RoBERTa based on Equation 6. The tuned RoBERTa has a parameter set θ_*hg*_, which maps *h*_*i*_ to *u*_*i*_ and *g*_*i*_ to *v*_*i*_ (see Equations 7 and 8), both of which are float tensors that quantify the sentiment tendency of *h*_*i*_ and *g*_*i*_, respectively.


(5) $$s_{i}=\{\begin{array}{ll} 0 & a_{i}=\text{No}\\ 1 & \text{otherwise} \end{array}$$

(6) $$\begin{array}{l} \theta_{hg}\leftarrow \underset{\theta }{\mathrm{argmin}}L\left[\left({\text{RoBERTa}}_{\theta }\left(h_{i}\right),s_{i}\right)+\left({\text{RoBERTa}}_{\theta }\left(g_{i}\right),s_{i}\right)\right] \end{array}$$

(7) $$\begin{array}{l} u_{i}={\text{RoBERTa}}_{\theta_{hg}}\left(h_{i}\right) \end{array}$$

(8) $$\begin{array}{l} v_{i}={\text{RoBERTa}}_{\theta_{hg}}\left(g_{i}\right) \end{array}$$

### 3.7. Training an XGBoost Classifier

Once *u*_*i*_ and *v*_*i*_ are generated for all samples in the training set, we store them in two sets $U={\left\{u_{i}\right\}}_{i=1}^{N}$ and $V={\left\{v_{i}\right\}}_{i=1}^{N}$, respectively. A sampling module is then employed to construct training samples for an XGBoost classifier. Specifically, we draw samples from *U* and *V* to obtain a collection of data points, denoted by $D^{UV}=\left\{\left[{\left(u_{a},v_{b}\right)}_{i},k_{i}\right]\right\}$, where *u*_*a*_ ∈ *U*, *v*_*b*_ ∈ *V*, and *k*_*i*_ is defined in Equation 9. In other words, *a* = *b* indicates that *u*_*a*_ and *v*_*b*_ are from the same abstract and should have the same sentiment tendency, making a positive example (i.e., *k*_*i*_ = 1); otherwise, we have a negative example (i.e., *k*_*i*_ = 0). We train an XGB classifier on *D*^*UV*^ (given in Equation 10). The trained XGB classifier takes an input (*u*_*i*_, *v*_*i*_) and outputs a confidence score *t*_*i*_ between 0 and 1, indicating the chance that *u*_*a*_ and *v*_*b*_ are generated from the same abstract.


(9) $$k_{i}=\{\begin{array}{ll} 0 & a\neq b\text{ for }{\left(u_{a},v_{b}\right)}_{i}\in D^{UV}\\ 1 & \text{otherwise} \end{array}$$

(10) $$\begin{array}{l} \theta_{uv}\leftarrow \underset{\theta }{\mathrm{argmin}}L\left[\left({\text{XGB}}_{\theta }\left({\left(u_{a},v_{b}\right)}_{i}\right),k_{i}\right)\right] \end{array}$$

(11) $$\begin{array}{l} t_{i}={\text{XGB}}_{\theta_{uv}}\left({\left(u_{a},v_{b}\right)}_{i}\right) \end{array}$$

### 3.8. Prediction

The previous steps have set up the models used for prediction. During inference, a question and the given context are fed into the T5 models to produce the short forms, and then converted to sentiment values, namely, *u* and *v*, by the RoBERTa model. Lastly, the XGBoost estimator takes as input *u* and *v* and outputs a confidence score *t*_*i*_ that determines the prediction result of the main learning task. Specifically, if *t*_*i*_ is larger than a given confidence threshold, we output *v*_*i*_ as a predicted answer for *q*_*i*_; otherwise, we add noise to the embedding of [*q*_*i*_; *h*_*i*_], and repeat the steps given by Equations 4, 8, and 11, to regenerate *t*_*i*_ until the confidence is larger than the threshold. The idea of adding noise to the word embeddings is from Wang et al. (2019). A similar procedure is adopted in our study. Specifically, the noise vector is sampled from a Gaussian distribution modeled on the word embeddings in the training data. We add the noise vector to [*q*_*i*_; *h*_*i*_] and divide the result by two to obtain a new embedding for the following steps. As discussed in Wang et al. (2019), this noise adding step potentially benefits the performance. The rationale is that the noise is generated from a Gaussian distribution modeled on the original training data, and adding such a noise helps diversify the data and thus serves as a test time augmentation strategy.

### 3.9. Performance Metric

We adopt the same performance metrics as Jin et al. (2019), including accuracy (Acc) and the F1 score. Due to the class imbalance issue, Acc does not sufficiently reflect the true performance since it may drive the learning algorithm to predict all samples as the major class label and can still obtain a high Acc. For our case, this could lead to many false negatives, i.e., the “No/Maybe” answers are misclassified to “Yes” answers. Therefore, F1 is adopted as a secondary metric. F1 is defined on top of precision (Pre) and recall (Rec). With the given true positives (TP), true negatives (TN), and false positives (FP), and false positive (FP) the definitions of Acc, Pre, Rec, and F1 can be given below.


(12) $$\begin{array}{l} Acc=\frac{TP+TN}{TP+FP+TN+FN}\times 100\% \end{array}$$

(13) $$\begin{array}{l} Pre=\frac{TP}{TP+FP}\times 100\% \end{array}$$

(14) $$\begin{array}{l} Rec=\frac{TP}{TP+FN}\times 100\% \end{array}$$

(15) $$\begin{array}{l} F1=2\times \frac{Pre\times Rec}{Pre+Rec}\times 100\% \end{array}$$

## 4. Experiments and Results

### 4.1. Implementation Details

The T5 small variant was adopted in this study due to its lightweight setting, with six transformer layers, eight-head attention, 512-dimensional word embedding, 2048-dimensional forward sublayer, resulting in 60M trainable parameters. All experiments were implemented using Python 3.6.7 and PyTorch 1.7.1 and conducted on a Windows 10 workstation with an i7-10875h CPU and a Tesla V100 16G GPU.

### 4.2. Hyperparameters

The hyperparameters used for training/fine-tuning the models are listed in Tables 3–5. For each model in the learning pipeline, we tune a subset of hyperparameters using a grid search. In addition, for the confidence threshold mentioned in Section 3.8, we also perform a linear search in the range of 0 and 1 with an interval of 0.1, and the best threshold used in the experiment is 0.4. The best combinations of hyperparameters were adopted to build the final model.

**Table 3:** Hyperparameter setting for T5 small.

**Hyperparameter**	**Tuned range**	**Opt**.
Weight decay	0.01	0.01
Dropout probability	0.1	0.1
Steps	20,000	20,000
Optimizer	Adam	Adam
Learning rate	[1E-2, 1E-3, 1E-4]	1E-3
Batch size	[8, 16, 32]	32

**Table 4:** Hyperparameter setting for RoBERTa.

**Parameters**	**Tuned range**	**Opt**.
Sequence length	128	128
Train batch size	[4, 8, 16]	16
Dev batch size	8	8
Test batch size	8	8
Learning rate	[1E-5, 3E-5, 1E-4, 3E-4]	1E-4
Epoch number	[3, 6, 9]	3
Warmup	0.1	0.1
Dropout	0.1	0.1

**Table 5:** Hyperparameter setting for XGB.

**Hyperparameter**	**Tuned range**	**Opt**.
eta	0.015	0.015
max_depth	[3, 5, 10]	5
n_estimator	[20, 40, 60]	20
sub_sample	0.5	0.5
scale_pos_weight	1.75	1.75
random_state	2	2
eval_metric	logloss	logloss
objective	binary:logistic	binary:logistic
num_round	50	50
test_frac	0.2	0.2

### 4.3. Benchmarks

Results from the prior two studies are used for comparison.

- **Multi-phase BioBERT** was developed by Jin et al. (2019), in which the PubMedQA dataset was created. The authors adopted a multi-phase fine-tuning strategy to tune the BioBERT model. Specifically, the model was sequentially fine-tuned on PQA-A, bootstrapped PQA-U, and finally PQA-L with either (Q, L) or (Q, C) as the input. Results showed that the multi-phase BioBERT outperformed other design alternatives.
- **BioELECTRA** (Kanakarajan et al., 2021) is a version of ELECTRA (Clark et al., 2020) but pretrained on the biomedical corpora. ELECTRA introduced a novel pre-training task, named replaced token detection (RTD), which was proposed to replace the mask language modeling (MLM) task adopted in BERT (Devlin et al., 2018). Instead of masking the input tokens, RTD pollutes the input by replacing some tokens with alternative ones sampled from a generator. Meanwhile, a discriminator is trained to predict whether or not each token in the polluted input sentence is a replaced one. RTD is more efficient and effective than MLM because the former predicts every token's genuineness rather than a subset of masked tokens. Therefore, the pre-trained models using RTD could generate contextual embeddings with higher quality, compared to BERT (Clark et al., 2020). BioELECTRA is obtained by pre-training ELECTRA from scratch on a large biomedical text collection that consists of PubMed abstracts and PMC full text articles. Based on Kanakarajan et al. (2021), BioELECTRA achieved SOTA on all of the 13 tasks in the BLURB benchmark (Gu et al., 2020), and PubMedQA was one of the datasets.

In addition, Jin et al. (2019) also reported the human performance evaluated from a single annotator.

### 4.4. Model Fine-Tuning

Table 6 reports the fine-tuning results for T5 based on Equation 1. T5 was fine-tuned with three epochs until the training and validation losses converge. We also adopted the ROUGE score to measure the quality of generated text (i.e., *c*_*i*_), compared against the target text (i.e., *l*_*i*_). Results show that all four ROUGE scores were over 90%, meaning that the tuned T5 model has learned to transform a context to its associated conclusion. Similar results can be found in Table 7, which shows the fine-tuning results for T5 based on Equation 3. It is noted that the effect was not as good as the previous task since the ROUGE scores are below 90, and the worst one, ROUGE-2, is only 71.19%. The results show that the second fine-tuning task is more difficult than the first one. After all, the model needed to learn how to map a long text (i.e., [*q*_*i*_; *h*_*i*_]) to a very short one (i.e., *a*_*i*_), and the underlying reasoning logic is difficult. Lastly, Table 8 shows the tuning results of RoBERTa based on Equation 6. It is observed that after two epochs, the model achieved a training Acc of 0.97.

**Table 6:** Fine-tuning T5 based on Equation 1.

**Epoch**	**T.L**.	**V.L**.	**ROUGE-1**	**ROUGE-2**	**ROUGE-L**	**ROUGE-Lsum**	**G.L**.
1	1.73	0.99	5.25	4.17	6.53	5.27	1.9
2	0.14	0.09	84.61	86.12	91.51	91.49	29.8
3	0.1	0.06	91.59	90.12	91.6	91.58	33.9

*T.L., training loss; V.L., validation loss; G.L., generated length*.

**Table 7:** Fine-tuning T5 based on Equation 3.

**Epoch**	**T.L**.	**V.L**.	**ROUGE-1**	**ROUGE-2**	**ROUGE-L**	**ROUGE-Lsum**	**G.L**.
1	0.23	0.23	82.25	67.19	82.24	82.19	11.41
2	0.23	0.21	84.69	70.5	84.68	84.68	7.46
3	0.18	0.2	85.27	71.19	85.26	85.25	4.44

**Table 8:** Fine-tuning RoBERTa.

**Epoch**	**T.L**.	**V.L**.	**Acc**	**Err. rate**
1	0.53	0.44	0.82	0.18
2	0.13	0.11	0.97	0.03

### 4.5. Ablation Study

Table 9 shows the results of ablation study that compares three design alternatives. The first model directly fine-tuned BioBERT on the PubMedQA dataset without using any sentiment information. The third model is the full setting of the proposed SentiMedQAer, and the second model removes the T5 module from the full setting. The job of T5 is to transform the long contexts and questions to their short forms, which is optional. It is observed that fine-tuning BioBERT resulted in the worst performance, with an Acc of 66.51% and an F1 of 52.48%, which is aligned with the result in Jin et al. (2019). The addition of sentiment information significantly boosts the performance, with a gain of 15.32% in Acc and 21.69% in F1 for the second model, compared to the fine-tuned BioBERT baseline. Moreover, we show that adding a T5 module to the second model can further boost the Acc and the F1 by 2.08% and 2.75%, respectively, meaning that T5 can extract essential knowledge from the otherwise long abstract and benefit the reasoning. We also report the inference speed for the three models in the last column of Table 9. The speed is measured by the average inference time (in second) per sample. It is noted that adding sentiment analysis into the pipeline incurs extra time (1.59 vs. 2.05), which is expected. However, the inclusion of T5 greatly reduces the inference time to 0.41 s, because T5 is trained to transform a long piece of text in the sample to a short form, only keeping the critical information. Thus, the sample size is reduced, which benefit the inference speed since less tokens need to be processed. This result also justifies the necessity of T5.

**Table 9:** Ablation study.

**Model**	**Acc**	**F1**	**Inference speed (s)**
Fine-tuned BioBERT	66.51	52.48	1.59
SentiMedQAer w/o T5	81.83	74.17	2.05
SentiMedQAer	83.91	76.92	0.41

### 4.6. Comparison With the SOTA

**Figure 2:**
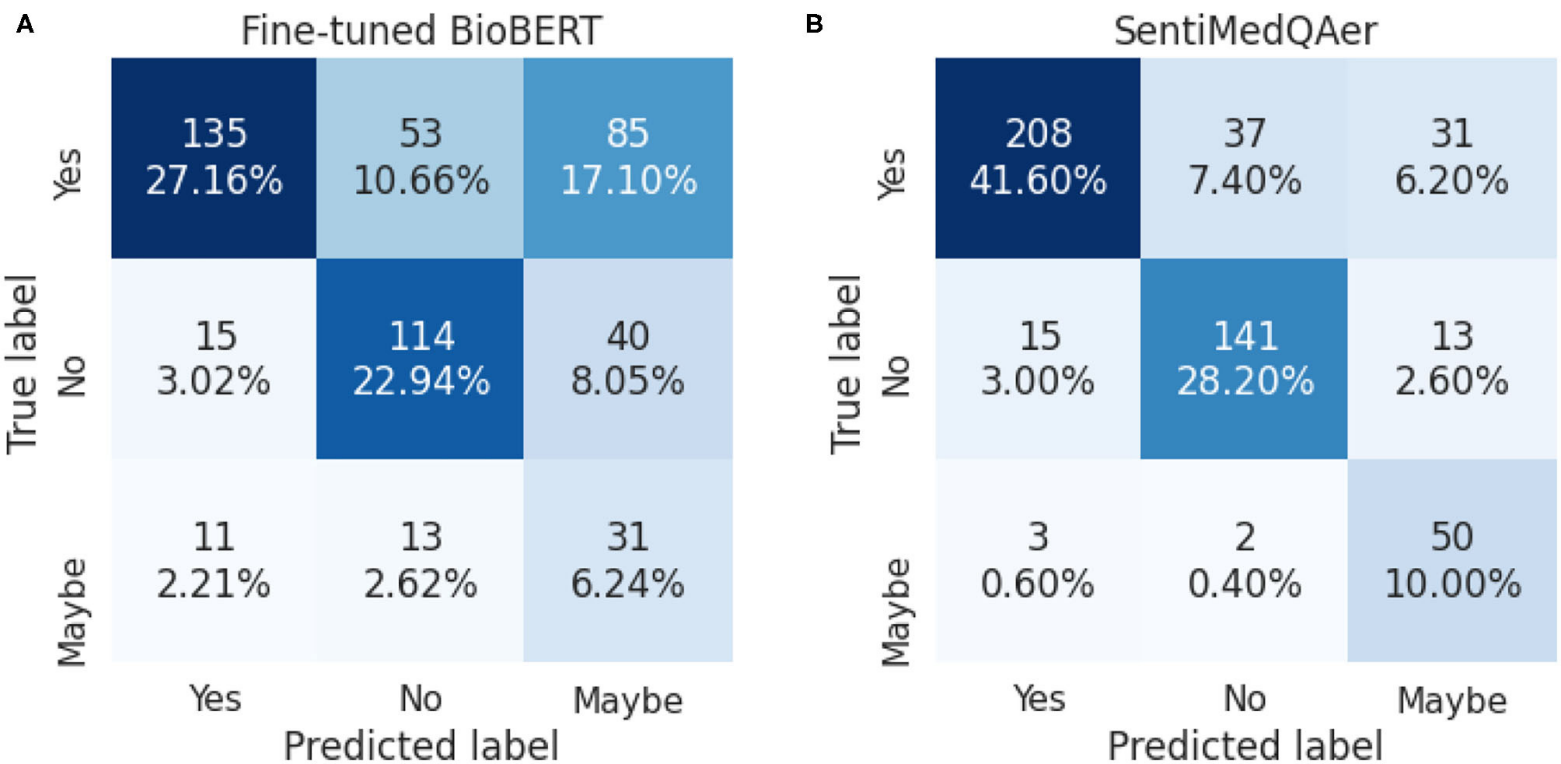
Confusion matrices for **(A)** the fine-tuned BioBERT and **(B)** SentiMedQAer.

We report a performance comparison between the proposed method and prior two studies in Table 10. It can be seen that the proposed SentiMedQAer model posted the best performance in both Acc and F1, outperforming the SOTA, multi-phase BioBERT, by 15.83% in Acc and 24.44% in F1, and also outperforming another strong baseline, BioELECTRA, by 19.69% in Acc. Figure 2 compares the confusion matrices of the fine-tuned BioBERT and the proposed SentiMedQAer on the test set. Compared to BioBERT, SentiMedQAer reduces the error rate by 14.44, 5.26, and 3.76%, for the categories of “Yes”, “No”, and “Maybe”, respectively. The results demonstrate the superiority of SentiMedQAer and validate the importance of sentiment tendency that can effectively boost the prediction performance.

**Table 10:** Comparison with the benchmarks.

**Model**	**Year**	**Acc**	**F1**
Fine-tuned BioBERT Jin et al. (2019)	2019	66.51	52.48
Single human performance Jin et al. (2019)	2019	78.0	-
Multi phase BioBERT Jin et al. (2019)	2019	68.08	52.72
BioELECTRA Kanakarajan et al. (2021)	2021	64.2	-
SentiMedQAer (ours)	2021	**83.91**	**76.92**

*The bold values indicate the highest score for each metric*.

### 4.7. Error Analysis

We conduct error analysis on the samples that are misclassified by SentiMedQAer. Two categories of errors have been observed. We provide our interpretation below.

- Several samples have shown both positive and negative statements in the long answer (i.e., the conclusion), which may confuse our model. For example, given a question “Is the zeolite hemostatic agent beneficial in reducing blood loss during arterial injury?” and a long answer “we observed that zeolite tends to reduce blood loss, however could not stop bleeding completely. We believe that further clinical trials are needed…”. The ground truth answer is “yes”, and our model predicted “no” due to the opposite sentiment tendencies expressed by words “tends to reduce” and “however could not”.
- Several samples with a ground truth answer “maybe” have also been misclassified. For instance, a sample with a question “Should chest wall irradiation be included after mastectomy and negative node breast cancer?”, a long answer “Post-mastectomy radiotherapy should be discussed for a sub-group of node-negative patients…”, and a context “Factors associated with increased risk of local failure were age < or = 40 years and tumor size greater than 20mm, without statistical significance.” For this case, our model predicted “no”, which may be due to the “without statistical significance” in the context.

Both error types are hard cases. To help the model improve on these cases, more similar samples are needed. However, in the original dataset, these cases are rare since most scientific findings are directly and explicitly described in the abstract. One potential way to address this issue is to employ generative models such as generative adversarial networks (Goodfellow et al., 2020) or GPT-3 (Floridi and Chiriatti, 2020) to create synthetic samples of these types.

## 5. Discussion

Transfer learning has recently been applied to numerous natural language processing (NLP) tasks, and question answering (QA) is one of them. Transfer learning allows a model to inherit domain knowledge obtained from an existing model that has been sufficiently pre-trained on a large domain-specific corpus in a self-supervised way. This study proposes a transfer learning-based sentiment-aware model, named SentiMedQAer, for biomedical QA. The proposed method consists of a learning pipeline that utilizes BioBERT to encode text tokens with contextual and domain-specific embeddings, fine-tunes Text-to-Text Transfer Transformer (T5) and RoBERTa models to integrate sentiment information into the model, and trains an XGBoost classifier to output a confidence score to determine the final answer to the question. We validate SentiMedQAer on PubMedQA, a biomedical QA dataset with reasoning-required yes/no questions. Results show that our method outperforms the SOTA by 15.83% and a single human annotator by 5.91%, demonstrating the effectiveness of SentiMedQAer in the task of yes/no biomedical QA. Sentiment in this study serves as an indicator for weakly supervised learning, which has become a popular learning paradigm recently. It is surprising that adding sentiment analysis into the model can boost the performance by a large margin, which demonstrates an important application of sentiment analysis as an auxiliary component in a learning framework. It is promising to see more applications of sentiment models in a wider range of NLP tasks.

The performance boost brought by SentiMedQAer can greatly improve the accuracy for medical QA applications, which can be extensively utilized by medical, clinical, and pharmaceutical researchers and practitioners on a daily basis. A typical use case is to build a QA engine in the medical domain. Users can send questions (which need to be transformed to yes/no questions) to the backend server, which hosts the a collection of models. The fist step is to find the most similar question stored in the database, and then SentiMedQAer can do its job and return an answer to the client user. Although popular search engines in the market are intelligent enough to answer generic questions, building a domain-specific QA engine is still of high demand and poses great challenges. SentiMedQAer offers one potential route to fulfill this need.

This study has the following limitations that will be addressed in future work. First, the proposed method should be validated in datasets other than PubMedQA. The reason why we chose PubMedQA as the learning task is that the short form of the answers, namely “yes/no/maybe”, clearly indicates the sentiment tendency. The idea of integrating sentiment information into the learning pipeline allows a model to use the sentiment as knowledge to guide the training. This philosophy can be utilized in other QA tasks or even a wider range of NLP tasks. It is a definite plan for us to keep exploring this direction. Second, it is desirable to reveal how sentiment can help boost the performance of a QA task in a more interpretable way, which is not sufficiently studied in this work. Also, to what degree the prediction of a QA sample can be explained by its associated sentiment remains to answer. Third, this study does not take data augmentation into account, which is worth further investigation. The creation of the PubMedQA was time-consuming since it is expensive to manually annotate the dataset, leading to a low-resource task. The PQA-A dataset that includes artificially generated answers was developed to address this problem. The efficacy of PQA-A has been verified. However, it would be interesting to explore other approaches to generate high-quality artificially labeled QA samples, especially for the hard cases mentioned in Section 4.7.

## Data Availability Statement

The dataset used to support this study is available at: https://github.com/pubmedqa/pubmedqa.

## Author Contributions

XZ, YC, YG, and ZX: conceptualization and methodology. XZ, YC, and YG: software, validation, and original draft preparation. XZ and ZX: review and editing. All authors have read and agreed to the published version of the manuscript.

## Conflict of Interest

The authors declare that the research was conducted in the absence of any commercial or financial relationships that could be construed as a potential conflict of interest.

## Publisher's Note

All claims expressed in this article are solely those of the authors and do not necessarily represent those of their affiliated organizations, or those of the publisher, the editors and the reviewers. Any product that may be evaluated in this article, or claim that may be made by its manufacturer, is not guaranteed or endorsed by the publisher.
